# pH‐driven entrapment of enrofloxacin in casein‐based nanoparticles for the enhancement of oral bioavailability

**DOI:** 10.1002/fsn3.2224

**Published:** 2021-04-07

**Authors:** Zhi‐xiang Yuan, Shichen Deng, Li Chen, You Hu, Jian Gu, Lili He

**Affiliations:** ^1^ College of Pharmacy Southwest Minzu University Chengdu China; ^2^ College of Animal & Veterinary Sciences Southwest Minzu University Chengdu China; ^3^ Department of Pharmacy College of Veterinary Medicine Sichuan Agricultural University Chengdu China

**Keywords:** casein, enrofloxacin, nanoparticles, oral bioavailability

## Abstract

Enrofloxacin (ENR), a broad‐spectrum antibacterial drug, has extremely poor water solubility contributing to low bioavailability, which prevents drug formulation design and limits its wide application in livestock farming and aquaculture. Compared to conventional formulations of ENR, casein (Cas)‐based drug delivery system has been reported to have significant advantages in the improvement of solubility and bioavailability of drugs. In this paper, we report the preparation process of ENR‐loaded Cas nanoparticles (ENR‐Cas) using magnetic agitation without any organic agent and the optimization of the formulation. Transmission electron microscopy (TEM), dynamic light scattering (DLS), differential scanning calorimetry (DSC), powder X‐ray diffraction (PXRD), and Fourier transform infrared spectroscopy (FTIR) were all adopted to characterize the ENR‐Cas. Results showed that the obtained ENR‐Cas were approximately spherical with a particle size of 171.6 ± 13.8 nm with a polydispersity index of 0.322 ± 0.053. In vitro release behavior of ENR‐Cas showed a sustained release profile. Additionally, in vivo study in rats displayed that the mean plasma concentration of ENR after oral administration of ENR‐Cas was significantly higher than that treated with ENR suspension. The mean residence time (MRT_0–24_) of ENR was enhanced by Cas nanoparticles from 9.287 ± 0.524 to 11.372 ± 1.139 hr in comparison with ENR suspension. Accordingly, the area under the curve (AUC_0–24_) of ENR‐Cas was 80.521 ± 6.624 μg·hr/ml, 3.8‐fold higher than that of ENR suspension (20.850 ± 1.715 μg·hr/ml). Therefore, it can be concluded that ENR‐Cas enhanced the absorption, prolonged the retention time, and improved oral bioavailability of ENR. Taken the good oral safety of Cas into consideration, ENR‐Cas should be a more promising oral preparation of ENR for clinical application.

## Graphical abstract

Herein, we report the preparation process of ENR‐loaded casein (Cas) nanoparticles (ENR‐Cas) without any organic agent and the optimization of the formulation. It can be concluded from the results that ENR‐Cas enhanced the absorption, prolonged the retention time, and improved oral bioavailability of ENR. Taken the good oral safety of Cas into consideration, ENR‐Cas should be a more promising preparation of ENR for clinical application.

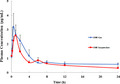

## 1 INTRODUCTION

With the rapid development of nanotechnology, many conventional areas of food science and food industry face to be revolutionized (He & Hwang, 2016). The research in developing food‐based nano‐encapsulated delivery systems is emerging. The demand of food‐based nanomaterials has been increased in the fields since many of them abound in essential elements, non‐toxic, and stable at high temperature and pressures. The food‐based nanostructured materials can be used as additives, carriers for smart delivery of bioactive compounds, or active agents to control the release and improve the bioavailability as well as nutritional value of food on the basis of their functions (Kaya‐Celiker & Mallikarjunan, 2012; Singh et al., 2017).

Casein (Cas), a food‐based natural polymer accounting for approximately 80% of the protein in milk, is considered a generally recognized‐as‐safe (GRAS) ingredient (Chen et al., 2019), which is widely used as vehicles for bio‐actives owing to its excellent properties (Livney, 2010; Pojanavaraphan et al., 2010). The composition of Cas involves four proteins known as α_1_, α_2_, β, and κ‐casein in approximate ratios of 4:1: 3.5:1.5 with unique structures and functions, which are di‐block copolymers with pronounced amphiphilic structure, allowing them to self‐assemble in water to form nanoscale core‐shell nanocomposites, where the hydrophobic blocks aggregate into the core and the hydrophilic blocks assemble the shell (Haratifar & Guri, 2017; Ma et al., 2015). Thus, drugs are able to enter the core of nanocomposites through physical embedding, electrostatic interaction, or covalent bonding (Liu et al., 2020; Pan et al., 2014). Another significant feature making Cas highly suitable for a drug carrier is its high composition of proline residues (17%), leading to an open tertiary structure, which is easily accessible to gastric proteases and releases the entrapped drug (Shapira et al., 2012). The oral bioavailability of folic acid loaded into Cas nanoparticles was promoted to be about 52%, which was 50% higher than conventional aqueous solutions (Penalva et al., 2015). Similar sustained release profile was observed in the report from Luo et al. (2015). In addition, Turovsky et al. (2015) have also developed β‐casein micelles as potential carriers in order to improve the poor water solubility of celecoxib for oral administration. Therefore, Cas nanoparticles could be considered as an ideal carrier to improve the solubility and dissolution properties of drugs and then enhance bioavailability.

Enrofloxacin (ENR), a second generation of fluoroquinolone antibiotics, has a broad spectrum of activity together with the capacity of acting against extracellular and intracellular infections, which has been used for oral and parenteral treatment of bacterial infections in animals. However, the extremely poor water solubility (0.23 g/L) of ENR leads to low bioavailability and puts a block on drug formulation design (Pei et al., 2020). In order to overcome the weaknesses of ENR, some researchers prepared different ENR nanocomposites using various carriers. For instance, Xie et al. prepared different ENR solid lipid nanoparticles (SLN) by three different fatty acids as lipid matrix and investigated the characteristics and pharmacokinetics of the SLN. The results presented that all three SLN observably improved the bioavailability of ENR and prolonged the average residence time after single intramuscular administration in mice (Xie et al., 2011), suggesting that the use of nanocarrier could be a smart way to promote the bioavailability of ENR. Nevertheless, the use of organic solvent, undefined safety, and non‐commercial availability of materials may have problems in limiting the application of the reported ENR nanoparticles.

There is a possibility that Cas can be exploited as a nanocarrier of hydrophobic drugs for protection of incorporated labile drugs from degradation, controlled release, and enhancing the bioavailability of hydrophobic drugs (Abhishek et al., 2008; Bar‐Zeev et al., 2016). In this study, we focused on optimizing the formulation and preparation process of ENR‐loaded casein nanocomposite (ENR‐Cas) without any addition of organic solvents by magnetic agitation and ultrasonication. The obtained ENR‐Cas was characterized using DLS, TEM, powder X‐ray diffraction (PXRD), DSC, and FTIR. In addition, in vitro release of ENR‐Cas and its pharmacokinetic behavior after oral administration in rats were also evaluated.

## 2 MATERIALS AND METHODS

### 2.1 Materials

Enrofloxacin (purity > 98%), ofloxacin (internal standard, purity > 99%), Cas, and pepsin were generously supplied by Sigma‐Aldrich. Methanol (HPLC grade) was purchased from Mreda Technology Co., Ltd. All other chemicals were of analytical grade.

### 2.2 Animals

Sprague Dawley rats (in male) weighing between 250 and 280 g were purchased from the Da‐Shuo experimental Animal Ltd. The animals were allowed to acclimatize for a few days in environmentally controlled quarters (24 ± 1°C, 12 hr light/dark cycle) and, unless specified otherwise, were provided with water and food ad libitum. All animal studies were approved by the Animal Ethical Experimentation Committee, College of Veterinary Medicine (SYXK(Chuan)2014‐187) and were performed according to the requirements of the People's Republic of China National Act on the use of experimental animals.

### 2.3 Preparation of ENR‐Cas

Enrofloxacin‐Casein was prepared as previously described by Pan et al. (2014) with minor modifications. A certain amount of Cas was dispersed into 20 ml of deionized water until dissolved. The Cas solution was adjusted to pH 12 using 4 M sodium hydroxide solution at room temperature and mixed by a magnetic stirrer for 30 min. Then, a certain amount of ENR powders was added into the Cas solution while being stirred for another 10 min, and then, the pH was adjusted to corresponding values with 4 M hydrochloric acid. The sample was subsequently homogenized with ultrasonication treatment under the condition of ice bath for a few minutes to yield a homogenous colloidal suspension. The factors influencing the formulation properties were screened by control variable method, including the amount of ENR, the concentration of Cas, the pH of ENR‐Cas solution, the ultrasonication power, and time in the system. The formulation and preparation method were optimized by evaluation of entrapment efficiency (EE) and loading efficiency (LE) as indexes using HPLC analysis. The ENR‐Cas was stored at 4°C after lyophilization in a freeze‐dryer (LyoQuest, Telstar) under 0.4 mbar vacuum for 24 hr and a condenser temperature of −40°C (Liu et al., 2020).

### 2.4 High‐performance liquid chromatography (HPLC) analysis

The concentration of ENR was determined at 277 nm (Agilent 1260 HPLC; Agilent C18 column [200 mm × 4.6 mm, 5.0 μm]; Agilent Technologies Inc.) and guarded with a precolumn at 30°C. Mobile phase consisted of 30% methanol and 70% formic acid solution (2%, v/v) (Kawas et al., 2018). The injection volume was 10 μl, and signals were collected at a flow rate of 1.0 ml/min. Method validation assays were carried out by selectivity, linearity, precision, and recovery, respectively.

### 2.5 Characterization of ENR‐Cas

#### 2.5.1 Determination of entrapment efficiency (EE) and loading efficiency (LE)

Ultrafiltration method was used to determine the entrapment efficiency and loading efficiency. The freshly prepared ENR‐Cas suspension was centrifuged at 1753 ×*g* (Sorvall ST16, Thermo Fisher Scientific) for 15 min in ultrafiltration tubes (MWCO = 3,000, Millipore Corporation) to separate the filtrate containing the non‐encapsulated ENR. Then, the filtrate was appropriately diluted with deionized water followed by the injection onto HPLC. The amount of non‐encapsulated ENR in the filtrate was determined by HPLC, and then, EE and LE were calculated by the following equations:

(1) $$\text{EE}\;\%=\frac{\left(\text{Total}\;\text{amount}\;\text{of}\;\text{ENR}‐\text{non - encapsulated}\;\text{ENR}\right)}{\text{Total}\;\text{amount}\;\text{of}\;\text{ENR}\times 100\%}$$

(2) $$\text{LE}\;\%=\frac{\left(\text{Total}\;\text{amount}\;\text{of}\;\text{ENR}‐\text{non - encapsulated}\;\text{ENR}\right)}{\left[\left(\text{Total}\;\text{amount}\;\text{of}\;\text{ENR}‐\text{non - encapsulated}\;\text{ENR}\right)+\text{Total}\;\text{amount}\;\text{of}\;\text{Cas}\right]\times 100\%}$$

#### 2.5.2 Particle size and ζ potential

The particle size and ζ potential of ENR‐Cas were measured at 25°C based on the dynamic light scattering and electrophoretic mobility principles using Malvern Zetasizer Nano ZS90 (Malvern Instruments Ltd). The samples were diluted to 100 times with purified water before determination. After equilibrating for 60 s, the samples were subjected to laser diffraction or Doppler velocimetry for the output of particles size and ζ potential.

#### 2.5.3 TEM image

The morphology of ENR‐Cas was inspected by using S4800 Transmission electron microscope (Hitachi Ltd). Briefly, the optimal ENR‐Cas was appropriately diluted in water and dripped onto a dedicated copper mesh and, then, negatively stained with 4% (w/v) phosphotungstic acid solution for 30 s. After drying at ambient temperature, nanoparticles were observed under Transmission electron microscope.

#### 2.5.4 DSC

Differential scanning calorimeter (Mettler Toledo) was performed for thermal analysis. Three to five milligram of samples (including ENR, Cas, physical mixture of ENR and Cas, and ENR‐Cas freeze‐dried powder) was used in each analysis and heated in a nitrogen atmosphere at a rate of 5°C/min to analyze the change of the sample within the temperature ranged from 30 to 350°C with an accuracy of temperature adjustment of +0.02°C, respectively.

#### 2.5.5 PXRD

Powder x‐ray diffractometer (AG‐10TA, Shimadzu) was also used for diffraction study. ENR, Cas, physical mixture of ENR and Cas, and ENR‐Cas freeze‐dried powder were, respectively, subjected to PXRD at a step size of 0.02° by exposing the samples to CuK_α_ radiation (40 kV, 200 mA) and scanning continuously from 0.5° (2θ) to 60° (2θ) with step time of 1 s.

#### 2.5.6 FTIR

Enrofloxacin, Cas, physical mixture of ENR and Cas, and ENR‐Cas freeze‐dried powder were also analyzed using infrared spectrometer (Perkin Elmer BX). About 5 mg dried samples were blended with solid potassium bromide (KBr) power, and then, the blend was tableted using a single‐punch tablet press and subjected to FTIR spectrometry. Transmittances were recorded at wave numbers of 4,000–400 cm^−1^ at a resolution of 4 cm^−1^ and scanning rate of 200 cm^−1^·s^−1^ by means of the Spectrum Time Bose Perkin Elmer program.

### 2.6 In vitro release of ENR‐Cas

In vitro release studies were carried out correspondingly in PBS (pH 7.4) and simulated gastric fluid (SGF, 3 mg/ml of pepsin was dispersed in saline and the pH was adjusted to 2.0 with concentrated HCl) using dynamic membrane dialysis. 1 ml of ENR‐Cas was added into a dialysis bag (3,500 Da) followed by dialysis against 99 ml PBS or SGF in 250‐ml beaker which was situated in oscillating water bath at a temperature of 37 ± 0.5°C. In predetermined time intervals (0.25, 0.5, 1, 2, 4, 6, 8, 10, 12, and 24 hr), 1 ml of release medium was withdrawn for determining the ENR diffused through the dialysis bag, and the same volume of fresh release medium preheated at 37°C was subsequently added into the beaker. Samples were analyzed by HPLC method as described previously. Cumulative drug release was calculated (Zu et al., 2016).

### 2.7 Oral bioavailability study

Before the experiment, 12 SD rats were randomly divided into two groups of six animals each for pharmacokinetic analysis. Animals were orally administrated with ENR suspension (ENR powders suspended in 2% HPMC solution (w/v)) and ENR‐Cas with an equivalent dose of 20 mg/kg ENR, respectively. 0.5 ml of blood samples was withdrawn from the ocular veniplex and placed in heparinized tubes at 5, 15, 30 min, 1, 2, 4, 6, 8, 12, and 24 hr. The blood samples were subsequently centrifuged at 4,000 rpm for 10 min (Sorvall ST16, Thermo Fisher Scientific) to prepare the plasma.

To quantify the plasma ENR, a liquid–liquid extraction procedure was applied to retrieve ENR from the plasma. In brief, 200 μl of plasma was mingled with 1.2 ml of dichloromethane, 100 μl of ultrapure water, and 100 μl of 200 μg/ml ofloxacin solution as an internal standard. After vortex for 2 min, the mixtures were centrifuged at 8,000 *g* for 10 min. The bottom liquid was transferred to new tubes and the extraction was repeated once. All collected organic extracts were pooled and dried under a gentle stream of nitrogen at 40°C, and then, the residues were reconstituted in 100 μl of methanol. After vortex mixing and centrifugation at 15777 ×*g* for 10 min, the supernatant was collected and then injected into HPLC system as described above, and the separation was performed on the C_18_ analytical column (4.6 × 250 mm, 5 μm) maintained at 30°C. Mobile phase consisted of 0.025 M phosphate acid (adjusted pH to 3.0 with triethylamine)/acetonitrile (17:83) with the flow rate of 1.0 ml/min (Kawas et al., 2018).

### 2.8 Statistical analysis

The DAS 2.0 (BioGuider Co) computer software was used to process the data and extract the pharmacokinetic parameters. Data were compared with the SPSS 16.0 statistical package. Multiple comparisons of mean values were performed by one‐way ANOVA with Fisher's least significant difference (LSD) test applied for post hoc comparisons at 95% confidence interval. *p* < .05 was considered statistically significant.

## 3 RESULTS

### 3.1 Characterization of ENR‐Cas

#### 3.1.1 Encapsulation efficiency

The HPLC method was applied for the determination of ENR successfully. The retention time of ENR was approximately 6.53 min. The calibration curves employed to calculate the concentration of ENR in the ultrafiltrate of ENR‐Cas were linear over the range of 10.0–80.0 μg/ml (*A* = 92,986*C* + 160,023, *A* and *C* stood for Peak area and concentration, respectively, *R* ^2^ = .9998). The recovery/accuracy was close to 100% for the three concentrations (20, 40, 60 μg/ml) tested. Additionally, the intra‐day and inter‐day precision of ENR was determined to be 0.44% and 0.57%, respectively.

**FIGURE 1:**
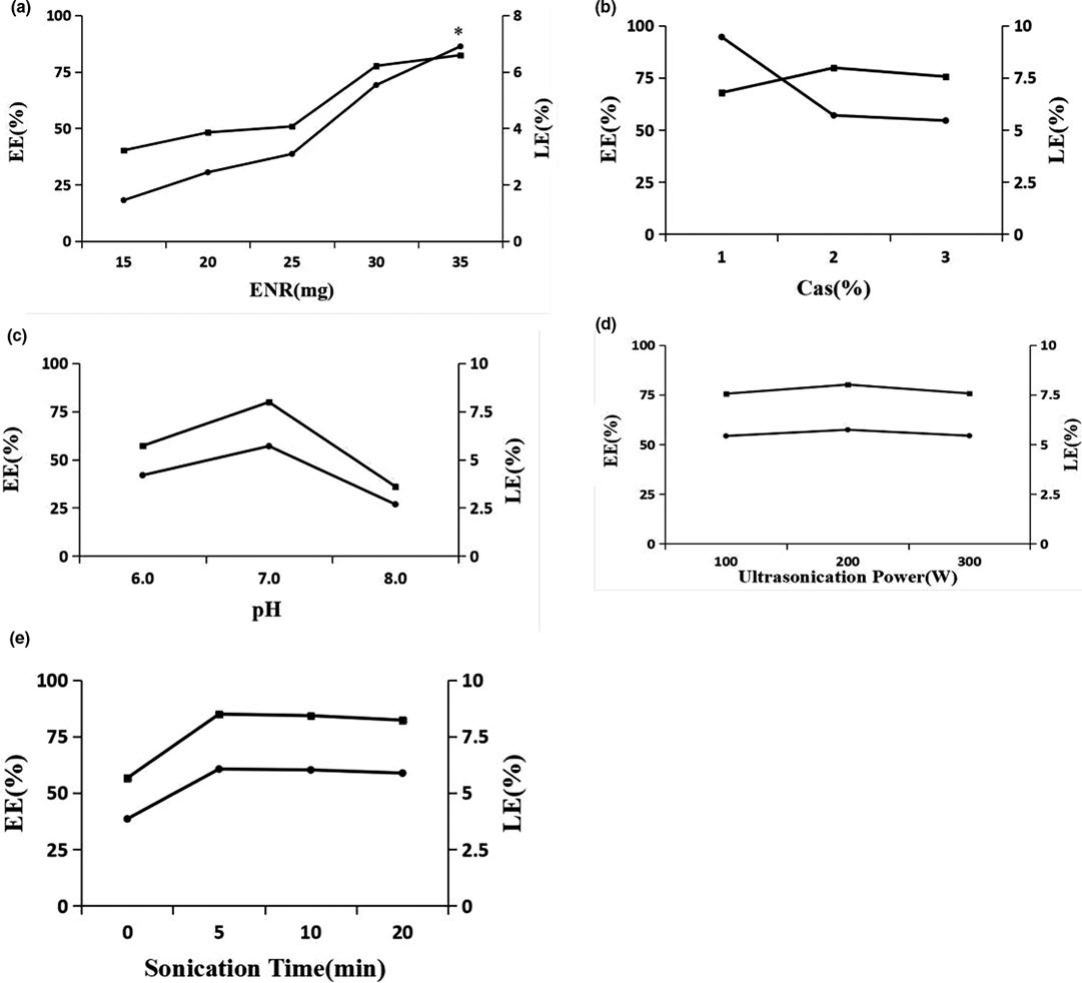
Formulation screening for the preparation of ENR‐Cas [five factors: the amount of ENR (a); the concentration of Cas (b); pH value (c); ultrasonication power (d); ultrasonication time (e)], *Means the obtained ENR‐Cas was instable

The effect of formulation variables on the EE and LE is shown in Figure 1. The result of different amount of ENR on the EE and LE demonstrated that the more amount of ENR in the formulation was, the higher EE of ENR‐Cas was (Figure 1a). However, 35 mg of ENR used in the formulation could cause the instability of ENR‐Cas suspension. As shown in Figure 1b, the EE increased with the increase of carrier concentration while the LE displayed a declining trend. To obtain a better EE of ENR, 2% w/w Cas should be selected as the optimal carrier concentration. In addition, pH value was adjusted to neutral (7.0) which leaded to the highest EE of ENR‐Cas (Figure 1c). There was no obvious effect on the EE and LE of ENR‐Cas by promoting ultrasonication power (Figure 1d). Extension of ultrasound time resulted in the increase of EE and LE initially, and then, a decreasing trend was observed, indicating the ideal ultrasonic time was 300 s (pulse on, 10 s; pulse off, 10 s) (Figure 1e). Therefore, the optimal formulation was as follows:

Four hundred milligram of Cas was dispersed into 20 ml of deionized water until dissolved. The Cas solution was adjusted to pH 12 using 4 M NaOH solution and mixed by a magnetic stirrer for 30 min. Then, 30 mg of ENR powders was added into the Cas solution while being stirred for another 10 min, and then, the pH was adjusted to 7.0 with 4 M HCl. The sample was subsequently homogenized with ultrasonication treatment (200 W, pulse on, 10 s; pulse off, 10 s) under the condition of the ice bath for 5 min to yield ENR‐Cas colloidal suspension.

#### 3.1.2 Particle size and potential

**FIGURE 2:**
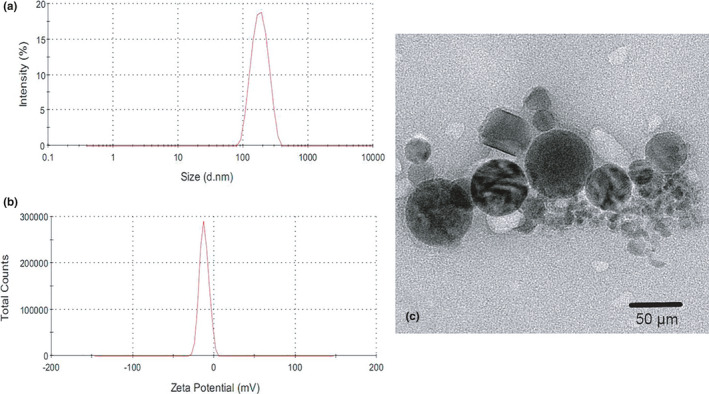
Size distribution (a), ζ potential distribution (b), and TEM image (c) of ENR‐Cas obtained using optimal formulation

The optimal ENR‐Cas colloidal suspension was obtained with a particle size of 171.6 ± 13.8 nm and a polydispersity index (PDI) of 0.322 ± 0.053 (Figure 2a). ENR‐Cas was negatively charged with a ζ potential of −12.1 ± 1.13 mV (Figure 2b), indicating that a high colloidal stability of ENR‐Cas due to the electrostatic repulsion. The physical stability of ENR‐Cas was shown to be fine for a short‐term storage survey. The significant changes of particle size and polydispersity index of ENR‐Cas did not occur. TEM results showed that ENR‐Cas appeared a nearly spherical morphology (Figure 2c).

#### 3.1.3 DSC

**FIGURE 3:**
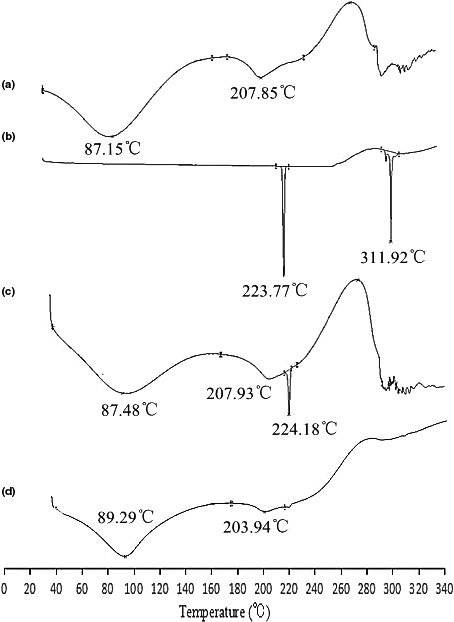
DSC thermograms of Cas (a), ENR (b), physical mixture (ENR and Cas, c) and ENR‐Cas (d)

The Cas, ENR, ENR‐Cas, and physical mixture of ENR and Cas have been studied by high sensitivity differential scanning calorimetry, and the results are shown in Figure 3. A wide endothermic peak of Cas at 87.2°C was caused by the dehydration of Cas while the endothermic peak at 207.8°C might be due to its melting (Figure 3a). An endothermic peak at 223.8°C representing the melting point of ENR and another sharp endothermic peak at 311.9°C corresponded to the flash point of ENR (Figure 3b). The physical mixture of ENR and Cas showed the characteristic peaks at 87.5, 207.9, and 224.2°C according to ENR and Cas. However, the characteristic peak of ENR only showed an exothermic peak with low intensity at 224.2°C in the thermogram of the physical mixture (Figure 3c), which could be induced by the decrease in crystallinity of ENR during the grinding and mixing of ENR and Cas. The lyophilized ENR‐Cas had a new endothermic peak at 203.9°C and no characteristic peak corresponding to melting point or flash point of ENR (Figure 3d), revealing that ENR was encapsulated in the Cas nanoparticles and converted to amorphous state from crystalline state.

#### 3.1.4 PXRD

**FIGURE 4:**
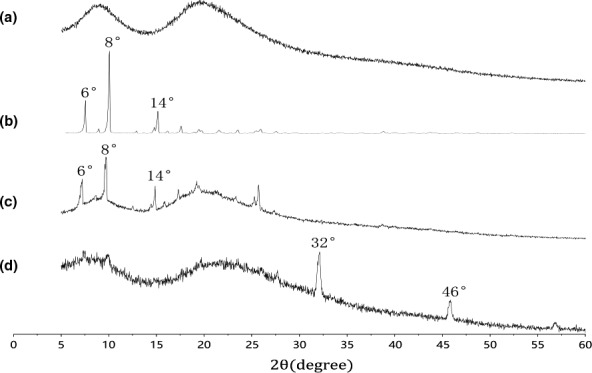
PXRD patterns of Cas (a), ENR (b), physical mixture (ENR and Cas, c), and ENR‐Cas (d)

The result PXRD patterns of samples are drawn in Figure 4. Cas had no diffraction characteristic peak, and only gentle and wide peaks were found in Cas diffractogram (Figure 4a) corresponding to its amorphous nature. However, strong sharp crystal diffraction peaks of ENR appeared in the range of 6–14° (Figure 4b), indicating that ENR was of typical crystalline nature. The PXRD of the physical mixture of ENR and Cas was considered a simple superposition of ENR and Cas (Figure 4c). Compared with the ENR diffractogram, the lyophilized ENR‐Cas presented amorphous pattern because there was no crystal diffraction peaks of ENR appeared in the range of 6–14° (Figure 4d). Notably, new crystal diffraction peaks appeared at 32°and 46°. Considering the use of PBS in the preparation, these crystal diffraction peaks may be attributed to NaCl crystal, which can be confirmed by the characteristic diffraction peaks at 27.4°, 31.7° and 45.4° and 56.4°in our previous report (Chen et al., 2020; Santo & Naldoni, 2019).

#### 3.1.5 FTIR

**FIGURE 5:**
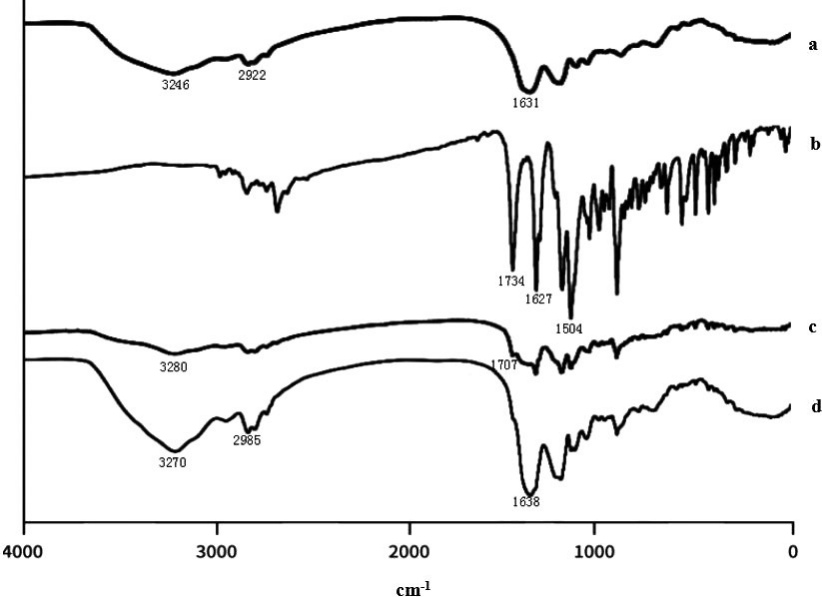
FTIR spectra of Cas (a), ENR (b), physical mixture (ENR and Cas, c), and ENR‐Cas (d)

The FTIR spectra of Cas, ENR, physical mixture (ENR and Cas), and ENR‐Cas are shown in Figure 5. The characteristic peaks of Cas were presented at 3,246 cm^−1^ (N‐H, O‐H stretching vibration), 2,922 cm^−1^ (C‐H stretching vibration), and 1,631 cm^−1^ (amide I stretching vibration) (Figure 5a). In the spectrum of ENR (Figure 5b), the characteristic peaks were detected at 1,734 cm^−1^ (C=O stretching vibration in carboxyl group), 1,627 cm^−1^ (carbonyl stretching vibration of 4‐pyridone) and 1,507 cm^−1^ (skeleton vibration of benzene ring). For the physical mixture, wider infrared bands at 3,280 and 1,707 cm^−1^ were observed, belonging to dual feature absorption of ENR and Cas (Figure 5c). The characteristic absorption of ENR‐Cas corresponding to OH bond stretching and carbonyl C=O slightly shifted to 1,771 and 3,406 cm^−1^, respectively. There was no obvious feature peak of ENR near 1,734 and 1,627 cm^−1^ in Figure 5d, which is evidence for successful entrapment of ENR into the nanoparticles.

### 3.2 In vitro release

**FIGURE 6:**
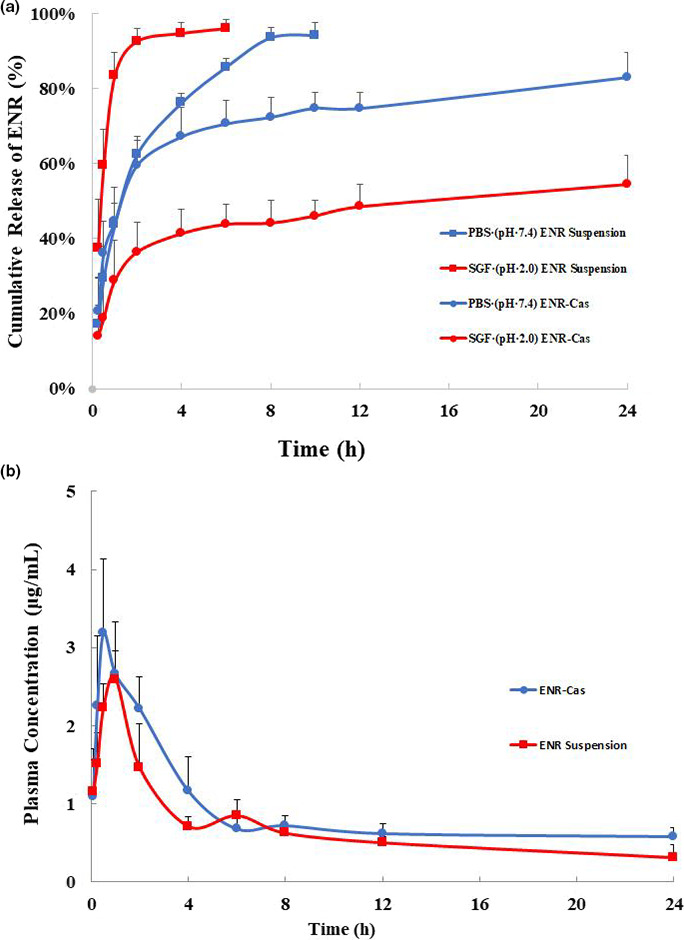
In vitro and in vivo studies. (a) In vitro release profiles of ENR suspension and ENR‐Cas in SGF (pH = 2.0, red) or in PBS (pH = 7.4, blue) using the dialysis bag diffusion technique. The experiment was repeated in triplicate, and data were given as the mean ± standard deviation (*n* = 3). (b) Plasma concentration‐time curves of ENR after oral administration of ENR suspension (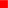) or ENR‐Cas (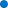) at a dose of 20 mg/kg to rats. Data were represented as the mean ± standard deviation (*n* = 6)

In vitro release behaviors of ENR suspension and ENR‐Cas in PBS (pH = 7.4) and SGF (pH = 2.0) are illustrated in Figure 6a. ENR suspension was rapidly released about 92.46% within 2 hr and completely within 4 hr under SGF condition, and the rate of release is significantly swifter than the release in PBS (pH = 7.4) due to the larger solubility of ENR in acid solution. The ENR‐Cas showed a biphasic drug release pattern in both PBS and SGF, which had an explosive release within the first 2 hr and then continuously released for 24 hr. However, it could be clearly seen that the release of ENR‐Cas in SGF (pH = 2.0) was lower than that of PBS (pH = 7.4).

### 3.3 Oral bioavailability study

The calibration curves for the determination of ENR level in plasma were linear over the range of 0.50–80.0 μg/ml (*A*′ = 0.025*C* − 0.0125, where *A*′ represents peak area of ENR normalized by an internal standard, *R* ^2^ = .9951). Herein, ofloxacin was used as an internal standard. Plasma concentration‐time profiles of ENR suspension and ENR‐Cas are shown in Figure 6b, and the pharmacokinetic parameters were summarized in Table 1. The *C*_max_ value for the ENR‐Cas (6.100 ± 0.974 μg/ml) was 2.6 times higher compared to the ENR suspension (2.292 ± 0.171 μg/ml). *T*_max_ for both groups were close (1.0 ± 0.021 hr for ENR suspension vs. 1.0 ± 0.037 hr for ENR‐Cas). Compared with the ENR suspension, the mean residence time (MRT_0–24_) of ENR was enhanced by Cas nanoparticles from 9.287 ± 0.524 to 11.372 ± 1.139 hr. Accordingly, AUC_0–24_ of ENR‐Cas was 80.521 ± 6.624 μg·hr/ml, 3.8‐fold higher than that of ENR suspension (20.850 ± 1.715 μg·hr/ml), indicating that ENR‐Cas enhanced the absorption, prolonged the retention time, and improved oral bioavailability of ENR.

**TABLE 1:** Pharmacokinetic parameters of ENR after oral administration for both group (20 mg/kg)

	*C*_max_ (μg/ml)	*T*_max_ (hr)	AUC_0–24_ (μg·hr/ml)	AUC_0–∞_ (μg·hr/ml)	MRT_0–24_ (hr)
ENR suspension	2.292 ± 0.171	1.0 ± 0.021	20.850 ± 1.715	67.671 ± 5.034	9.287 ± 0.524
ENR‐Cas	6.100 ± 0.974*	1.0 ± 0.037	80.521 ± 6.624*	493.637 ± 45.608*	11.372 ± 1.139*

Note

AUC_0–24_, the area under the concentration‐time curve from zero to 24 hr; AUC_0–∞_, the area under the concentration‐time curve from zero to infinity; *C*_max_, the maximum concentration of ENR in plasma; MRT, the mean residence time; *T*_max_, the time to reach *C*_max_.

* *p* < .05, significant difference compared to ENR suspension.

## 4 DISCUSSION

It is reported that ENR as a veterinary antibiotic has a broad spectrum of activity together with the capacity of acting against extracellular and intracellular infections, which has been used for oral and parenteral treatment of bacterial infections in animals. Nevertheless, the clinical use of ENR is often discouraged due to its poor water solubility and low bioavailability. Thus, how to enhance its solubility and improve bioavailability are key issues to be solved by developing new preparations. Various nanoparticle carriers have been developed to ameliorate the shortcomings of ENR; however, the high cost of encapsulation materials, limited availability, uncertain biocompatibility, and residual organic solvents prevent them from being used for industrial peer review (Tao et al., 2019). Cas is deemed as a GRAS ingredient, which not only has ideal gelling property, emulsifying property, and water binding property but also has wide applicability in drug release. On top of mentioned above, the Cas has the ability of self‐assembling to form nanoparticles in the water that mainly depends on temperature, ionic strength, and protein concentration (Pan et al., 2014). Therefore, Cas is chosen as the carrier in this study to load ENR by using a simple method for overcoming its shorts with the hopes of providing valuable information for the combination of ENR and Cas, and opening up new and exciting ways for the preparation of ENR.

ENR‐Cas is successfully obtained by direct addition of ENR solution to the aqueous Cas solution without any organic solvents at ambient temperature under mildly magnetic stirring and then further homogenized by ultrasonication. The obtained ENR‐Cas shows the characteristics of nanoparticle size, good stability, and no organic solvent residue. The concentration of Cas is a crucial influence factor on particle size, EE, and LE of ENR‐Cas because of its viscosity property. Previous literatures manifested that Cas solution with low viscosity could promote the breakage of nanoemulsion droplet and reduce the load efficiency of the sample whereas high concentration was more viscous to promote precipitation of the sample, resulting in larger nanoparticle sizes (Li et al., 2017). Casein molecules can self‐assemble into casein micelles in the pH ranges from 5.5 to 12.0 mainly through hydrophobic interaction, hydrogen bond, and electrostatic interaction, and the structure of the micelle is more compact at low pH and looser at high pH (Shapira et al., 2012). In this study, Cas solution with pH of 12 was adjusted to be neutral after addition of ENR; then, the structure of Cas changed from “relaxed status” to “compact status,” which was beneficial to the formation of ENR‐Cas.

The obtained ENR‐Cas was redispersed into deionized water, and then, a clear colloidal solution was obtained for the measurement of particle size. As shown in Figure 2a, the average particle size of the optimal ENR‐Cas was 171.6 ± 13.8 nm and ENR‐Cas was approximately spherical with a uniform size distribution under TEM observation (Figure 2c). However, the diameter of ENR‐Cas observed from TEM seemed smaller than the data shown in Figure 2a. A possible reason might be due to the shrinkage of ENR‐Cas owing to drying of the sample during preparation for microscopy analysis. Besides, the zeta potential and PDI value of ENR‐Cas exhibited that ENR‐Cas is well dispersed and stable in water. The value of zeta potential illustrates the difference in charge between outer ions and bulk of the liquid surrounding the nanoparticles, which interprets that the large repulsion force between the nanoparticles could refrain from the flocculation and aggregation of the nanoparticles, maintaining a stable colloidal system. The zeta potential of ENR‐Cas obtained by using optimal formulation was −12.1 ± 1.13 mV (Figure 2b), which was consistent with the data from the previous report (Penalva et al., 2015). Cas nanoparticles are of colloidal size, which can be described as supramolecules consisting of multiple molecular entities held together and organized by means of non‐covalent intermolecular binding interactions. The negative charge of nanoparticles was probably attributed to the supramolecular structural changes of Cas above their isoelectric point.

To characterize ENR‐Cas, DSC, PXRD, and FTIR were performed on Cas, ENR, the physical mixture powder, as well as lyophilized ENR‐Cas. The DSC spectrum of ENR showed characteristic endothermic peak at 223.8 and 311.9°C while no typical endothermic peak was observed for ENR‐Cas. The loss of characteristic endothermic peak of ENR indicated resultful internalization of ENR in Cas nanoparticles, ascertaining effective combination between ENR and Cas (Gandhi & Roy, 2019). The PXRD pattern of ENR‐Cas also showed that the characteristic peak of ENR disappears in comparison to the crystalline form of ENR and the amorphous pattern of Cas. Furthermore, no peaks of crystalline ENR were observed in ENR‐Cas, revealing that ENR was encapsulated in the hydrophobic core of Cas in an amorphous form. In the FTIR diagram of ENR‐Cas, the characteristic absorption peaks of ENR and Cas were shifted and weakened, illustrating that the interaction between Cas and ENR (intermolecular hydrogen bonds) in the nanoencapsulation process leaded to changes in ENR group environment.

The release pattern of ENR from ENR suspension and ENR‐Cas were studied at two different release media: aqueous HCl with pepsin (pH = 2.0) to mimic gastric fluid and PBS (pH = 7.4) to mimic intestinal condition (Figure 6a). The release of ENR‐Cas displayed similar biphasic drug release patterns and slower release compared to the ENR suspension, which suggested that ENR‐Cas would be a promising controlled release preparation. Notably, the release pattern of ENR from ENR‐Cas was found to be dependent on the pH conditions. Only around 40% of ENR released from the nanoparticles in simulated gastric condition for 24 hr. This phenomenon might be attributed to the fact that pepsin preferentially attacks peptide bonds containing hydrophobic aromatic amino acids, which would be trapped inside Cas nanoparticles during the preparation of ENR‐Cas, preventing the release of ENR from Cas nanoparticles. Under simulated intestinal conditions (pH 7.4), ENR‐Cas presented a biphasic drug release pattern with a burst release within the first 2 hr and a sustained release thereafter, which was substantially consistent with the Cas nanoparticles loaded with flutamide (Elzoghby et al., 2013) and curcumin (Pan et al., 2014) in PBS (pH 7.4). This phenomenon might be related with the repulsion effect between the negative charges of ENR and casein at pH 7.4, which would eject ENR from the nanocarrier (Penalva et al., 2015). Thus, the release of ENR from ENR‐Cas reached more than 80% within 24 hr to ensure the adequate absorption of ENR in intestine. In addition, the initial burst release of ENR‐Cas would be helpful for the timely achievement of therapeutic concentration, which is important for the therapeutic efficacy of concentration‐dependent ENR. The following slow release would maintain effective therapeutic concentration in vivo.

In in vivo pharmacokinetic study, the plasma concentration of ENR after administration of ENR‐Cas reached the peak concentration within an hour, after which the concentration maintained above 0.1 μg/ml for a longer time than that of ENR suspension. We noticed that *T*_max_ for both groups were the same. The possible reason should be that ENR suspension is inherent in sustained release characteristics due to its poor dissolution compared to ENR solution. For both groups of ENR‐Cas and ENR suspension after oral administration, the plasma concentration‐time profiles presented a double‐peak, indicating that there was a hepatoenteral circulation in the metabolism of rats (Kaartinen et al., 2010). Underlying the fact that fluoroquinolones perform in a concentration‐dependent manner, the AUC_0–24_/MIC_90_ ratio is one of the best parameters for predicting their antimicrobial effect. ENR‐Cas remarkably improves the *C*_max_, AUC_0–24_, AUC_0–∞_, and MRT_0–24_ of ENR. Thus, the plasma concentration of 0.1 μg/ml is deemed to be therapeutically adequate (Xie et al., 2011). All the results demonstrate that ENR‐Cas possesses favorable bioavailability. The possible reason for the enhanced bioavailability might be that ENR‐Cas nanoparticles slowly released after entering the intestinal condition, which was helpful for the sufficient uptake of ENR in intestine. Moreover, it is so conceivable to reduce the frequency of administration within a certain period, which will be more advantageous to the application of ENR in veterinary.

In conclusion, we successfully prepared ENR‐Cas using a self‐assembly method in water. The optimal ENR‐Cas was characterized by DLS, TEM, DSC, PXRD, and FTIR analysis. Furthermore, in vitro release behavior showed a sustained release of ENR from ENR‐Cas nanoparticles, which guaranteed the absorption of ENR. In vivo pharmacokinetics study showed that ENR‐Cas enhanced the absorption, prolonged the retention time, and improved the oral bioavailability of ENR. Taken the good oral safety of Cas into consideration, ENR‐Cas should be a promising preparation of ENR for clinical application.

## CONFLICT OF INTEREST

The author(s) declared no potential conflicts of interest with respect to the research, authorship, and/or publication of this article.

## Data Availability

The data that support the findings of this study are available on request from the corresponding author. The data are not publicly available due to privacy or ethical restrictions.
